# Attitudes and Perceptions of School Teachers in Melilla Regarding the Care Provided to Students with Type 1 Diabetes

**DOI:** 10.3390/children8121137

**Published:** 2021-12-05

**Authors:** Trinidad Luque-Vara, Elisabet Fernández-Gómez, Marta Linares-Manrique, Silvia Navarro-Prado, María Angustias Sánchez-Ojeda, Carmen Enrique-Mirón

**Affiliations:** 1 Department of Nursing, Faculty of Health Sciences, Melilla Campus, University of Granada, Calle Santander s/n, 52001 Melilla, Spain; triluva@ugr.es (T.L.-V.); elisabetfdez@ugr.es (E.F.-G.); silnado@ugr.es (S.N.-P.); maso@ugr.es (M.A.S.-O.); 2 SEJ-658 Laboratory for Cognition, Department of Nursing, Health, Training and Interaction among Humans, Animals and Machines, Faculty of Health Sciences, Melilla Campus, University of Granada, Calle Santander s/n, 52001 Melilla, Spain; 3 HUM-613 Research Group, Department of Inorganic Chemistry, Faculty of Health Sciences, Melilla Campus, University of Granada, Calle Santander s/n, 52001 Melilla, Spain; cenrique@ugr.es

**Keywords:** type 1 diabetes, quality of life, prevention, schools, hypoglycaemia

## Abstract

The main objective of the study was to assess the perception of non-university teachers in the city of Melilla to help students with type 1 diabetes mellitus (T1DM), as well as their attitudes towards helping these students in diabetic emergencies. This observational, descriptive, cross-sectional study analyzed the answers given by 441 teachers from 25 public institutions in the city of Melilla to a survey on the attitude and perception regarding the capacity of educational institutions (16 questions) to help and manage students with T1DM. Out of 47.6% of teachers who represent having had students with TIDM, only 4.8% acknowledged having been trained in diabetes. The percentage that has experienced a hypoglycaemia episode at the institution was 29.9%. More than half of participants acknowledged that their educational institution is not prepared to manage diabetic emergencies. Only 5.7% stated their institutions have glucagon in their first-aid kit and less than half of participants (44.7%) would be willing to administer it if necessary. Teachers of educational institutions believe they have not been particularly trained in the care of students living with T1DM and point out that their educational institutions are not prepared to help in diabetic emergencies.

## 1. Introduction

There is an increase of Type 1 Diabetes Mellitus (T1DM) both in incidence and in prevalence [1]. At a worldwide level, it represents 10% of total cases, and it has always been, and still is, one of the most common chronic diseases in childhood. It is assumed that the number of children and adolescents with T1DM, younger than 20 years old, amounts to 1,110,100, and the number of new cases per year, to 128,900. In the last decade, an annual increase of approximately 3% of adolescents younger than 15 years old with this pathology [2] has been observed in many countries. In Spain, the evolution in the number of children and adolescents with type 1 diabetes is around 15,470 cases [3]. In the Autonomous City of Melilla, 26 people died of this condition in 2018 [4].

According to the *Standards of Medical Care in Diabetes*, an optimal glycaemic control is recommended in order to delay the appearance of long-term complications, such as retinopathy, nephropathy, and neuropathy, since these may be considered the three main causes of comorbidity in T1DM patients [5]. Diabetes care must be supervised by qualified people, not only within the family, but also within the educational environment. Children and adolescents spend many hours at school and T1DM must be monitored 24/7 in order to reduce any potential complications associated with the disease and to ensure students can safely participate in all school activities [6,7,8,9]. 

Recent studies show that diabetic emergencies, such as hypoglycaemia and hyperglycaemia, are quite frequent in the educational environment [9,10,11] and that is why children and adolescents with T1DM need to check their blood sugar level on a frequent basis, apart from administering insulin injections, and complying with diet and physical activity recommendations [12]. Still, there is a possibility of having a hypoglycaemia episode at non-controlled instances [13]. 

Ideally educational environments have immediate access to supplies to support students with diabetes, as well as personnel training in diabetes care [14,15]. Education and support are essential to help people self-manage the disease. Training is an ongoing process to achieve the necessary skills, capacities and knowledge for the successful management of the disease [16].

Despite the recommendations on a safe environment for the care of children with T1DM insufficient research has been conducted regarding the level of preparation of educational institutions and the attitudes of teachers towards supporting children and adolescents [17,18,19]. Furthermore, today at a national level, there is an insufficient number of school nurses able to provide medical attention at all times. Due to this, most parents and children would like school personnel to be prepared to manage T1DM [20], especially in emergency cases, so that they should be adequately informed and have a good level of knowledge about the disease [21].

Smith et al. outlined the need to develop educational programs to train school personnel in diabetes. It is important to conduct research to assess the perceptions of teachers’ institution’s preparation and their training to act in case of diabetic emergencies [20]. 

The main objective of the study was to assess the perception of teachers about the level preparation of public and state-subsidized private pre-school, primary, and secondary schools in the Autonomous City of Melilla to help students with T1DM, as well as teacher attitudes towards helping these students in diabetic emergencies.

## 2. Materials and Methods

### 2.1. Study Design

An observational, descriptive, cross-sectional study has been conducted to assess the attitudes and perception of teachers regarding the capacity of educational institutions in the City of Melilla to help students with T1DM.

### 2.2. Participants

The subjects of this study comprise non-university school teachers teaching at public and state-subsidized private institutions in the Autonomous City of Melilla. All participants included in the study have face to face contact with students. The total number of pre-school and primary school institutions was 16, and 9 were secondary educational institutions, amounting to 1500 teachers (Ministry of Education and Vocational Training, Melilla). Selection of the sample was performed by means of a non-probability, convenient sampling. A total of 441 teachers have participated in this research, the sampling error being 5%, with a confidence level of 95%. Those who did not sign the informed consent and those on leave at the time of data collection were excluded.

### 2.3. Instrument

The instrument administered to assess attitudes and perceptions of teachers regarding the capacity of the institution to provide care to students with T1DM is an adaptation of the questionnaire designed by Carral-San Laureano [13]. This questionnaire has two parts. The first part assesses the attitude and perception of teachers regarding the level of preparation of educational institutions to provide care to stu-dents with T1DM. Out of the total 16 items forming this part, 12 have three answer options (yes/no/don’t know) and the remaining 4 are open questions or questions with different answer options (Annex 1). The second part considers issues related to sociodemographic and educational aspects such as age, gender, years of experience as a teacher, type of teacher, presence in the classroom of T1DM students in the current year or in previous academic years, and qualifications.

In order to assess teacher perceptions of their institution’s capacity, those answering in the affirmative to the question “Do you think your institution is capable of managing diabetic emergencies?” were considered. A diabetic emergency was defined as a hy-poglycaemia episode (glucose < 70 mg/dL) and a hyperglycemic episode (glucose > 300 mg/L) during school hours, regardless of other related symptoms. Affirmative answers to the question “Would you be willing to administer glucagon if necessary?” were considered.

### 2.4. Study Procedure

For access to educational institutions, the approval of the Provincial Education Directorate of Melilla was obtained, as well as authorization of education institutions’ direc-tors. Once the teachers provided informed written consent, participants self-completed the questionnaire on an anonymous basis to ensure confidentiality. Data collection took place during the 2019/2020 academic year.

### 2.5. Data Analysis

Collected data were codified and entered into the study database and analyzed using the statistical program Statistical Package for the Social Sciences for Windows version 25.0 (SPSS v25.0). For the descriptive analysis, basic statistics were employed (frequencies and percentages for qualitative variables, and mean, standard deviation, median and range, for quantitative variables). The Chi-square test was utilized, based on contingency tables, to determine the statistical independence of the variables under study. All signif-icance values refer to bilateral tests, taking the statistically significant association into account if *p* < 0.05.

### 2.6. Ethical Considerations

The study has followed the guidelines and ethical principles of the Declaration of Helsinki. Participants were informed of the objective of the study, their participation was voluntary and they accepted and signed the participant information and consent form. This study is derived from the research project “Assessment of knowledge about diabetes in non-university teachers of the Autonomous City of Melilla as a health promotion practice” approved by the research commission of the Faculty of Health Sciences of Melilla, belonging to the University of Granada with registration number: 201700800000670. It was also approved by the Provincial Director of the Ministry of Education and Vocational Training in Melilla.

## 3. Results

Participating teachers had a mean age of 44.36 ± 9.5 years, (min 28-max 68). As depicted in Table 1, 17.7% (78 teachers) acknowledge they currently teach students with T1DM, and 47.6% (210 teachers) stated they have taught them in previous years. However, only 4.8% indicated receiving some kind of training in diabetes management (Table 2). With respect to diabetic emergencies, 29.9% of participants knew of at least one hypoglycaemia episode at the institution, and more than half (52.4%) believed their institution was not prepared to provide needed care to students who have diabetic emergencies. Only 5.7% stated that their institution has glucagon in their first-aid kit (29.7% think there is no glucagon and the remaining 64.4% did not know/did not reply). 39.2% felt there were no personnel prepared for glucagon administration. Nevertheless, if necessary, 44.7% would be willing to administer it (Table 2). 

In the univariate analysis of factors related to favorable perceptions of teachers (Table 3), it is evidenced that teachers with less than 10 years experience, as well as those who have had students with T1DM in previous academic years, show a more favorable perception of the institution than their co-workers. Secondary education teachers and, teachers with less than 10 years experience, show a more favorable attitude towards providing needed care to students with diabetes compared with teachers with over 10 years of experience or those teaching at other educational levels. In this respect, teachers with a favorable perception towards the capacity of the institution are generally younger than teachers with an unfavorable perception (41.03 ± 9.80 years olds vs. 46.49 ± 8.83 years old, *p* = 0.038, respectively). As regards the attitude towards administering glucagon, younger teachers were more likely to have a positive attitude, but, no significant differences were noted (42.40 ± 9.34 years old vs. 46.78 ± 9.25 years old, *p* = 0.792, respectively). In relation to the remaining variables analyzed, no significant differences were observed in the perception and atti-tudes of teachers towards the institution (Table 3).

## 4. Discussion

It is worth noting the high rate of cases of children from Melilla with type 1 diabetes, 15–16% more than the national mean. There is literature that evidences that T1DM is a multifactorial endocrine disorder. However, the cause of this high percentage of diabetic children in Melilla could be due to the particularities of the population. Consanguinity is a possible factor, which increases the prevalence of diabetes in citizens, more specifically in children, given that Melilla is a city barely open to foreigners, especially the Berbers (predominant population in Melilla), there are many marriages between distant cousins, thus promoting such consanguinity [22,23,24,25].

It is important to take into account whether educational centres are equipped and have qualified personnel to provide care to students with diabetes, so that they can be safe and fully included in different school activities [7,24]. The relevance of the aforementioned is supported by the perceptions of the study participants, given that a high percentage represented that diabetic students can participate in every school activity. 

The American Diabetes Association (ADA), has recently prepared several documents related to the management of diabetes in children and adolescents at educational institutions [26].

It is the responsibility of educational institutions to provide adequate training to school personnel regarding the, management and treatment of T1DM [27,28], the aims would be to assist with optimal metabolic control [29], bring about higher trust and satisfaction on the part of parents and students within the school environment [30], and improve quality of life of students with T1DM [31]. In line with the study carried out by Carral et al. [13], it is worth mentioning that students with T1DM would benefit from improved teacher knowledge, by decreasing their level of anxiety when facing diabetes emergencies and knowing their teachers are prepared to manage their disease. That is why it is recommended that public administrations should adopt T1DM programs targeting the entire school community, to all school teachers in general, and Physical Education teachers in particular, due to the impact of physical activity on glycaemic control; low blood glucose levels (hypoglycaemia) or higher levels (hyperglycaemia) [9,13,24]. Such programs are key mechanisms in the increase in support and management of the disease within the educational context.

Being aware that the only initial, approved therapy for severe hypoglycaemia is administration of glucagon [22,32], it is essential that institutions have this in their first-aid kits, appropriate care and supervision of students living with T1DM should be provided, especially with respect to glucose level and diabetic emergencies [9]. A high percentage of our participants stated being unaware of whether the institution had glucagon in their first-aid kit. There are other similar studies, which indicate that most parents felt there were limited glucagon injections at school, and the personnel prepared to administer glucagon were insufficiently trained [33,34,35]. The lack of specific equipment for the cooling of glucagon at schools, the non-availability of glucagon and the absence of qualified personnel at schools, along account an with an increase in students with T1DM be seen as dangerous since severe hypoglycemia associated with T1DM may lead to convulsions, coma and, eventually, death [10,28,29]. 

Only a fourth of teachers participating in this study indicated that their institution was prepared to manage diabetic emergencies.

It should be noted that a significant percentage of young teachers are willing to be trained in T1DM management. 

Although many teachers felt the preparation of the educa-tional institution was insufficient, they were personally willing to help care of T1DM students despite having received no specific training in the management of the disease. 

There are some limitations to our study. Firstly, the sample collected is not totally representative of all educational institutions and levels, due, mainly, to the restrictions of educational institutions because of the Covid-19 pandemic. Moreover, the lack of previous studies on this topic has been quite significant, since it has represented a problem when it comes to discussing and comparing it to other studies, but, in turn, it is an opportunity to identify new “cracks” in literature and, therefore, new knowledge is contributed by means of this study.

## 5. Conclusions

Finally, it is worth noting the perception about the inadequate training of participating teachers to provide needed care to T1DM students, as well as the lack of preparation of institutions to manage diabetic emergencies. In this sense, it is essential that the institutions involved implement training programs to prepare teachers to help diabetic students, as well as to evidence the need of reorganising human and material resources available at educational institutions with the aim of facilitating an appropriate care of T1DM students.

## Figures and Tables

**Table 1:** Characteristics of teachers included in the study (*n* = 441).

Variables		Frequency (%)
Gender (*n*,%)		
	Male	138 (31.3)
	Female	303 (68.7)
Experience as a teacher (*n*,%)		
	≤10 years	128 (29.0)
	>10 years	313 (71.0)
Qualifications (*n*,%)		
	Pre-school education	58 (13.2)
	Primary education	228 (51.7)
	Secondary education	155 (35.1)
Teacher type (*n*,%)		
	Director	10 (2.3)
	Counsellor	7 (1.6)
	Physical Education teacher Other teachers	31 (7.0) 393(89.1)
Teachers currently teaching students with T1DM (*n*, %)		78 (17.7)
Teachers who have previously taught students with T1DM (*n*, %)		210 (47.6)

**Table 2:** Answers to the questionnaire Attitudes and perception of teachers regarding the capacity of the educational institution to provide needed care to students with diabetes (*n* = 441, frequencies and percentages).

	Yes	No	Don’t Know/No Reply
Does the institution have any support from a nurse?	188 (42.6)	219 (49.7)	34 (7.7)
Are there at the institution any specific measures to help students with diabetes?	117 (26.5)	176 (39.9)	148 (33.6)
Has the personnel of the institution received specific training in diabetes and its control?	21 (4.8)	340 (77.1)	80 (18.1)
Have you experienced a hypoglycaemia episode at the institution?	132 (29.9)	292 (66.2)	17 (3.9)
Have any of these episodes taken place during or after physical education classes?	42 (9.5)	100 (22.7)	299 (67.8)
Do you think your institution is prepared to treat diabetic emergencies?	57 (12.9)	231 (52.4)	153 (34.7)
Does the institution have glucagon in its first-aid kit?	25 (5.7)	131 (29.7)	285 (64.6)
Does the institution have anyone prepared to administer glucagon?	36 (8.2)	173 (39.2)	231 (52.4)
Would you be willing to administer glucagon if necessary?	197 (44.7)	142 (32.2)	102 (23.1)
Are physical education teachers trained to recognise a hypoglycemic episode?	81 (18.4)	53 (12)	307 (69.6)
Can a student with diabetes participate in every school activity?	354 (80.3)	17 (3.8)	70 (15.9)
Are special measures taken for students with T1DM during school activities?	137 (31.1)	59 (13.4)	245 (55.6)

**Table 3:** Univariate analysis on attitudes and perceptions of teachers about their and their institutions´management of T1DM.

	Positive Perception of the Institution ^a^	*p*	Positive Attitude of the Institution ^b^	*p*
Male (*n* = 138)	17 (12.3)	0.933	71 (51.4)	0.106
Female (*n* = 303)	40 (13.2)		126 (41.6)	
Experience as teacher ≤ 10 years (*n* = 128)	20 (15.6)	<0.001	66 (51.6)	0.023
Experience as teacher > 10 years (*n* = 313)	37 (11.8)		131 (41.9)	
Teacher currently having T1DM students (*n* = 78)	11 (14.1)	0.719	38 (48.7)	0.201
Teacher not having T1DM students currently (*n* = 363)	46 (12.2)		159 (43.8)	
Teachers with T1DM students in previous academic years (*n* = 210)	29 (13.8)	<0.001	92 (43.8)	0.318
Teacher with no T1DM students in previous academic years (*n* = 231)	28 (12.1)		105 (53.3)	
Pre-school Ed. teachers (*n* = 58)	4 (6.9)	0.182	12 (20.7)	0.002
Primary Ed. teachers (*n* = 228)	38 (16.7)		109 (47.8)	
Secondary Ed. teachers (*n* = 155)	15 (9.7)		76 (49.0)	
Physical Education teachers (*n* = 31)	6 (19.4)	0.394	13 (41.9)	0.432
Other teachers (Physical Ed. excluded) (*n*= 410)	51 (12.0)		184 (45.0)	
School counsellors (*n* = 7)	1 (14.3)	0.507	3 (42.9)	0.220
Non-school counsellors (*n* = 434)	56 (13.0)		194 (45.0)	
Directors (*n* = 10)	2 (20.0)	0.246	5 (50.0)	0.236
Non-directors (*n* = 431)	55 (13.0)		192 (45)	

^a^ Teachers answering positively to the question of whether their institution is prepared to control diabetic emergencies. ^b^ Teachers answering positively to their willingness to administer glucagon if necessary.

## Data Availability

The data are not publicly available because the work presented is intended to be carried out in other educational centers in other cities.
